# *MED12* mutations in breast phyllodes tumors: evidence of temporal tumoral heterogeneity and identification of associated critical signaling pathways

**DOI:** 10.18632/oncotarget.12991

**Published:** 2016-10-31

**Authors:** Marick Laé, Sophie Gardrat, Sophie Rondeau, Camille Richardot, Martial Caly, Walid Chemlali, Sophie Vacher, Jérôme Couturier, Odette Mariani, Philippe Terrier, Ivan Bièche

**Affiliations:** ^1^ Service de Pathologie, Institut Curie, 75248 Paris Cedex 05, France; ^2^ Service de Génétique, Unité de pharmacogénomique, Institut Curie, 75248 Paris Cedex 05, France; ^3^ Service de Pathologie, Institut Gustave Roussy, 94805, Villejuif Cedex, France

**Keywords:** MED12, phyllodes tumors

## Abstract

Exome sequencing has recently identified highly recurrent *MED12* somatic mutations in fibroadenomas (FAs) and phyllodes tumors (PTs). In the present study, based on a large series, we confirmed the presence of *MED12* exon 1 and 2 mutations in 49% (41/83) of PTs, 70% (7/10) of FAs and 9.1% (1/11) of fibromatoses. We show that *MED12* mutations are associated with benign behavior of phyllodes tumors, as they are detected less frequently in malignant PTs (27.6%) compared to benign (58.3%) and borderline (63.3%) PTs, respectively (*p* = 0.0036). Phyllodes tumors presented marked temporal heterogeneity of *MED12* mutation status, as 50% (3/6) of primary and recurrent phyllodes tumor pairs with *MED12* mutation presented different *MED12* mutations between the primary and recurrent tumors. There was no correlation between MED12 status and genomic profiles obtained by array-CGH. *MED12* mutations are associated with altered expressions of the genes involved in the *WNT* (*PAX3, WNT3A, AXIN2*), *TGFB* (*TAGLN, TGFBR2, CTGF*) and *THRA (RXRA, THRA)* signaling pathways.

In conclusion, this study confirmed that *MED12* plays a central oncogenic role in breast fibroepithelial tumorigenesis and identified a limited number of altered signaling pathways that maybe associated with *MED12* mutations. *MED12* exon 1 and 2 mutation status and some of the altered genes identified in this study could constitute useful diagnostic or prognostic markers, and form the basis for novel therapeutic strategies for PTs.

## INTRODUCTION

Phyllodes tumors (PTs) and fibroadenomas (FAs) are fibroepithelial tumors of the breast. FAs are the most common form of fibroepithelial tumors of the breast (97.5%), while PTs account for 2.5% of fibroepithelial tumors of the breast and 0.5% of all breast tumors [1]. The peak incidence of PTs is observed in women between the ages of 35 and 55 years. PTs are biphasic tumors comprising a double-layered benign epithelial component and an overgrowing mesenchymal component. PTs mainly develop *de novo*, but cases of progression from FAs have been reported, in view of the histological similarities between these two tumors [1]. PTs are classified into 3 categories, benign, borderline, and malignant based on stromal atypias, stromal cellularity, stromal overgrowth, mitotic count and appearance of the margins according to the 2012 WHO classification [1]. This histological variability results in difficulties distinguishing prognostically reliable categories. Most PTs are benign (70%) with a 13% risk of recurrence. Fifteen percent are borderline with a 20% risk of recurrence. Malignant tumors (15%) with a 26% risk of recurrence may give rise to hematogenous metastasis [1]. It is therefore important to identify this last subgroup. Standard treatment is surgery, while the efficacy of adjuvant chemotherapy remains controversial.

The genetic changes responsible for initiation and progression of PTs have been poorly characterized. Some studies have provided insight into the molecular pathogenesis of PTs. The most commonly reported chromosomal copy number alterations in PTs identified by comparative genomic hybridization (CGH) or array CGH are 1q gain, 13q, 6q and 9p losses, but their prognostic significance is unclear [2–4]. Gene amplifications involving *MDM2, MDM4, RAF1, PDZD2, MYC, EGFR, IGF1R, TERT* have been reported in isolated cases of borderline and malignant PTs [3–5, 8]. Regions of homozygous deletion include the *CDKN2A* gene at 9p21.3 and the *MACROD2* gene at 20p12.1 [3, 4]. In addition, recurrent somatic mutations have been described in several genes, such as *RB1 and NF1* [5, 6], *FBX4*, *TP53* [7, 8], *RARA, FLNA, SETD2, KMT2D, BCOR*, *MAP3K1, PIK3CA, ERBB4, EGFR* [6], and *MED12*, which is the mutated gene most commonly associated with PTs.

Highly recurrent *MED12 (Mediator complex subunit 12)* somatic mutations have been identified in fibroepithelial tumors: in as many as 60% of breast FAs [6, 10] and 70% of breast PTs [5, 6, 10, 11]. The high frequency of *MED12* recurrent mutations suggests a central role of *MED12* mutations in the pathogenesis of FAs and PTs. *MED12* is located on Xq13.1 and is composed of 45 exons, although all *MED12* mutations described in tumors to date mainly affect exon 2 with mutation hotspots in codons 36 and 44, and more rarely affect exon 1. *MED12* mutations have also been reported in other tumor types such as hematological cancers (chronic lymphoid leukemia) [12], uterine leiomyomas [13, 14], and uterine leiomyosarcomas [15]. Analysis of 1,862 samples of major cancer types including sarcomas, colon, breast, and lung carcinomas and gastrointestinal stromal tumors, showed *MED12* mutations exclusively in 52.2% (35/67) of uterine leiomyomas except for 0.3% (1/389) of colon carcinomas [16]. The high prevalence of these *MED12* mutations in fibroepithelial tumors suggests that MED12 is a critical driver gene in fibroepithelial tumorigenesis. *MED12* encodes a co-transcriptional factor (a Mediator complex subunit) thought to facilitate bridging of DNA regulatory sequences to the RNA polymerase II initiation complex [17], that regulates RNA polymerase II-mediated transcription, thereby playing a role in cell development and survival [18]. The kinase/CDK8 module formed by MED12, CDK8/CDK19, Cyclin C and MED13 is often associated with transcriptional repression and is a coregulator within the p53 transcriptional program [1195]. As a transcriptional co-regulator, MED12 has also been implicated in the modulation of Wnt [20, 21, 17], sonic hedgehog [1822] and TGF-ß pathway signaling [1923].

In order to determine whether *MED12* mutation is a driver genetic alteration that can contribute to the formation of these fibroepithelial tumors, we investigated the incidence of *MED12* mutations in a large series of fibroepithelial tumors: PTs (*n* = 97, 83 primary PTs of all grades: 13 recurrences and 1 metastasis), FAs (*n* = 10) and fibromatosis (*n* = 11), a fibroblastic/myofibroblastic tumor of intermediate potential, which constitutes a differential diagnoses of fibroepithelial tumors.

We evaluated the spatial and temporal heterogeneity of *MED12* status, as well as correlations between *MED12* mutation status and clinicopathological features, CGH array genomic profile data, expression of genes known to be altered in tumorigenesis and genes involved in various steps of *MED12* signaling in human embryogenesis and other types of *MED12*-associated cancers.

## RESULTS

**Figure 1:**
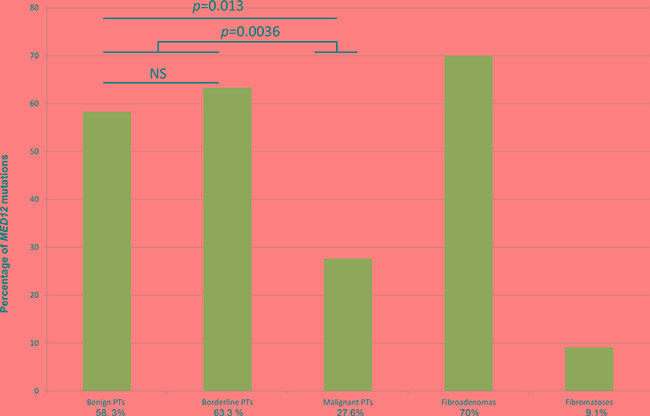
*MED12* mutation status in PTs (according to grade), FAs and fibromatosis

We studied *MED12* exon 1 and 2 mutations in a series of 83 primary PTs (24 benign, 30 borderline and 29 malignant), 10 FAs and 11 cases of fibromatosis. This analysis confirmed a strikingly high frequency of *MED12* exon 1 and 2 mutations in 41/83 PTs (49.4%) and 7/10 FAs (70%), but in only 1/11 cases of fibromatosis (9.1%). *MED12* mutations were identified with a high frequency in 14/24 (58.3%) benign PTs and 19/30 (63.3%) borderline PTs and more rarely in 8/29 (27.6%) malignant PTs (Table 1, Figure 1). Malignant PTs harbored *MED12* exon 2 somatic mutations significantly less frequently than the group of benign and borderline PTs (*p* = 0.0036) (Table 1). No significant associations were observed between *MED12* mutation and the patient's age, tumor size, mitotic count (*p* = 0.052) and second event (Table 1).

**Table 1:** Associations between *MED12* mutation status and clinicopathological features of 83 primary PTs

	Total population (%)	*MED12* wild-type PTs (%)	*MED12* mutated PTs (%)	*p*-value^a^
***Total***	83 (100)	42 (50.6)	41 (49.4)	
**Age**				
≤ 45	39 (47.0)	23 (59.0)	16 (41.0)	0.15 (NS)
> 45	44 (53.0)	19 (43.2)	25 (56.8 )	
***Macroscopic tumor size***				
≤ 50 mm	42 (50.6)	19 (45.2)	23 (54.8 )	0.32 (NS)
> 50 mm	41 (49.4)	23 (56.1)	18 (43.9)	
***Histological grade***				
Benign	24 (28.9)	10 (41.6)	14 (58.3)	Benign and borderline vs malignant: 0.0036
Borderline	30 (36.1)	11 (36.7 )	19 (63.3 )	Benign vs borderline vs malignant: 0.013
Malignant	29 (34.9)	21 (72.4)	8 (27.6)	Benign vs borderline and malignant: 0.29 (NS)
***Mitoses/10 high power fields***				
0–4	36 (43.4)	15 (41.7)	21 (58.3)	0.052 (NS)
5 to 9	16 (19.3)	6 (37.5)	10 (62.5)	
≥ 10	31 (37.3)	21 (67.7)	10 (32.2)	
***Second event***				
No second events	69 (83.1)	35 (50.7)	34 (49.3 )	0.58 (NS)
Recurrence	13 (15.7)	6 (46.2)	7 (53.8)	
Metastasis	1 (1.2)	1 (100)	0 (0)	

a Chi-square test.

NS: not significant.

**Figure 2:**
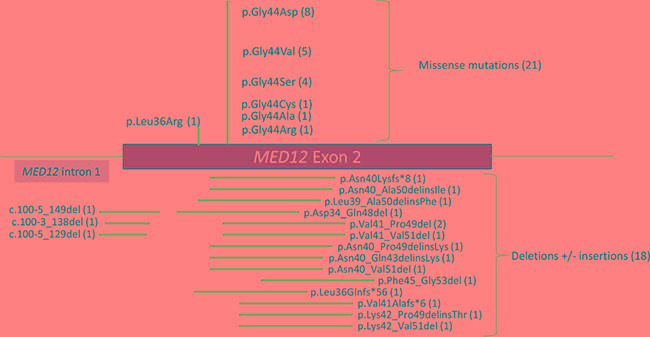
Diagram showing the distribution of *MED12* exon 2 mutations detected in this study The number of each alteration is indicated in parentheses.

The *MED12* mutational spectrum observed in these 83 PTs was mainly composed of point substitutions in 21 tumors (25.3%) and deletions (+/− insertions) in 20 tumors (24.1%) (Supplementary Table S1). Point substitutions mainly consisted of missense mutations substituting the Gly residue in codon 44 (8 p.Gly44Asp, 5 p.Gly44Val, 4 p.Gly44Ser, 1 p.Gly44Cys, 1 p.Gly44Ala, 1 p.Gly44Arg) in 20 tumors 24.1%) and in codon 36 (p.Leu36Arg) in one tumor (1.2%). Additionally, twelve (14.4%) samples harbored deletions (+/− insertions) within *MED12* exon 2 presumably designed to preserve the reading frame, and another three (3.6%) samples harbored a frameshift deletion. Deletions encompassing a part of intron 1 and the beginning of exon 2 were detected in three samples (3.6%) (Figure 2). Two samples harbored deletion within *MED12* exon 1 (2.4%).

There was no difference between the type of mutations (eg., missense vs deletion) in malignant/borderline PTs vs FAs/benign PTs (*p* = 0,14) (chi square test, data not shown)

In order to determine whether *MED12* exon 1 and 2 mutations are somatic mutations in PTs, we evaluated *MED12* status on 10 samples with non-neoplastic tissue from the 41 lesions harboring a *MED12* mutation. None of these non-neoplastic tissue samples harbored mutations, indicating the somatic nature of the *MED12* mutations in PTs.

We correlated *MED12* mutations with follow-up (available for all 83 PTs). Fourteen patients presented a second event (13 recurrences and 1 metastasis). Second events occurred in 4/24 benign, 7/30 borderline and 3/29 malignant PTs. One of the 7 borderline PTs presented a malignant skin nodule on the chest wall and a pelvic subcutaneous metastasis. All benign and borderline PTs were treated by lumpectomy, while malignant PTs were treated by mastectomy. Among the 14 tumors with second event 7 primary tumors harbored *MED12* mutations and 7 were wild-type. *MED12* mutation was not associated with disease-free survival (*p* = 0.51) (Supplementary Figure S1). In the group of 30 borderline PTs, no significant differences in terms of disease-free survival were observed between WT or mutated *MED12* tumors (*p* = 0.74, data not shown)

Mutational status was available for ten of the fourteen samples of second events: four pairs of primary and recurrent PTs were *MED12* wild-type and six were *MED12* mutated (Table 3). Three of the six *MED12* mutated pairs of primary and recurrent PTs harbored identical mutations. Three pairs (P1/R1, P2/R2 and P6/R6) presented distinct mutations in the primary and recurrent tumors, highlighting the temporal heterogeneity of these tumors (Table 2).

**Table 2:** *MED12* mutation status between primary tumor and second event (recurrence or metastasis)

Primary tumor				Second event			
Name	Grade	CDS mutation	AA mutation	Name	Grade	CDS mutation	AA mutation
**P1**	Benign	c.131G > A	p.Gly44Asp	**R1**	Benign	c.130G > T	p.Gly44Cys
**P2**	Benign	c.131G > A	p.Gly44Asp	**R2**	Borderline	c.130G > T	p.Gly44Cys
**P3**	Benign	c.122_148del	p.Val41_Pro49del	**R3**	Benign	c.122_148del	p.Val41_Pro49del
**P4**	Benign	WT	WT	**R4a**	Benign	WT	WT
				**R4b**	Benign	WT	WT
				**R4c**	Benign	WT	WT
**P5**	Borderline	c.131G > A	p.Gly44Asp	**R5**	Borderline	c.131G > A	p.Gly44Asp
**P6**	Borderline	c.121_153del	p.Val41_Val51del	**R6**	Benign	c.130G > T	p.Gly44Cys
**P7**	Borderline	c.132_158del	p.Phe45_Gly53del	**R7a**	Borderline	c.132_158del	p.Phe45_Gly53del
				**R7b**	Borderline	c.132_158del	p.Phe45_Gly53del
				**R7c**	Borderline	c.132_158del	p.Phe45_Gly53del
				**R7d**	Borderline	c.132_158del	p.Phe45_Gly53del
**P8**	Borderline	WT	WT	**R8**	Borderline	WT	WT
**P9**	Borderline	WT	WT	**M9**	Malignant	WT	WT
**P10**	Malignant	WT	WT	**R10**	Malignant	WT	WT

Abbreviations: CDS: coding sequence; AA: amino acid; P: primary tumor; R: recurrence; WT: wild type; M: metastasis.

**Table 3:** Relationship between *MED12* mutation status and CGHarray data in a series of 53 PTs

	Total population (%)	*MED12* wild-type PTs (%)	*MED12* mutated PTs (%)	*p*-value^a^
***Total***	53 (100)	27 (50.9)	26 (49.1 )	
***Median number of CNA***		3.0 (0–20)	3.0 (0–16)	0.36 (NS) ^b^
**1q gain +**	21 (39.6)	12 (57.2)	9 (42.8)	0.69 (NS)
**1q gain −**	32 (60.4)	16 (50.0)	15 (50.0)	
**3q loss +**	7 (13.2)	5 (71.4)	2 (28.6)	0,59 (NS)
**3q loss −**	46 (86.8)	24 (52.2)	22 (47.8)	
**6q loss +**	11 (20.8)	7 (63.6)	4 (36.4)	0.34 (NS)
**6q loss −**	42 (79.2)	20 (47.6)	22 (52.4)	
**7p gain +**	12 (22.6)	7 (58.3)	5 (41.7)	0.77 (NS)
**7p gain −**	41 (77.4)	22 (53.7)	19 (46.3)	
**8q gain +**	8 (15.1)	5 (62.5)	3 (37.5)	0,92 (NS)
**8q gain −**	45 (84.9)	24 (53.3)	21 (46.7)	
**10p loss +**	16 (30.2)	8 (50)	8 (50)	0.92 (NS)
**10p loss−**	37 (69.8)	19 (51.4)	18 (48.6)	
**10q loss +**	6 (11.3)	5 (83.3)	1 (16.7)	0.29 (NS)
**10q loss−**	47 (88.7)	24 (51.1)	23 (49.9)	
**13q loss +**	16 (30.2)	8 (50.0)	8 (50.0)	0.69 (NS)
**13q loss−**	37 (69.8)	19 (51.4)	18 (48.6)	

a Chi-square test.

b Kruskal Wallis's H test.

CNA: copy number alterations.

NS: not significant.

Two patients with PTs presented multiple metachronous recurrences (R4 a,b,c and R7 a,b,c,d); all recurrent lesions harbored identical *MED12* somatic mutations (Table 2).

To evaluate intratumoral spatial heterogeneity, we studied different blocks from the same sample (primary tumors P1, P6 and recurrent tumors R1, R6). All blocks from the same sample harbored identical *MED12* somatic mutation profiles (data not shown).

In order to determine whether *MED12* mutations are associated with particular genomic profiles, we compared our array-CGH data [24] for 53 borderline (*n* = 30) and malignant (*n* = 23) PTs with *MED12* mutation status. Malignant PTs have a trend to to harbor more chromosomal number alterations than borderline PTs (median of 5 versus 1.5) (*p* = 0.0054; Kruskal Wallis test). Among borderline and malignant tumors, the genomic profiles of the 26 *MED12*-mutated PTs and the 27 *MED12*-WT tumors have the same median number of chromosomal number alterations CNAs = 3 (Kruskal Wallis's H test, *p* = 0.36) (Table 3). Frequent recurrent imbalances (> 10% of cases with CNAs) were gains of entire 1q, 7p, 8q and losses of 3q, 6q, 10p, 10q and 13q. These eight alterations were not significantly associated with *MED12*-WT or *MED12*-mutated tumors (Table 3). Within each group of borderline PTs and malignant PTs, the mean number of CNAs and the most common recurrent imbalances were not significantly correlated with *MED12* status (data not shown).

In the group of ten FAs samples, three tumors harbored point mutations in codon 44 (1 p.Gly44Asp, 1 p.Gly44Ser, 1 p.Gly44Val), three tumors harbored deletions or insertions that were presumably designed to preserve the reading frame (p.Asn47_Ser52del, p.Thr37_Val51del, p.Phe45delinsCysVal) and one tumor harbored a frameshift deletion (p.Val41Serfs*6). One of the eleven cases of fibromatosis sequenced harbored point mutation in codon 44, p.Gly44Glu.

MED12 immunohistochemistry was performed on a series of 57 PTs with known *MED12* status (27 *MED12*-mutated tumors and 30 *MED12*-WT tumors) in order to evaluate MED12 protein expression. Nuclear expression of MED12 protein was observed in the stromal cell component of 46/57 PTs (80.7%) with preferential localization of nuclear-positive stromal cells around epithelial structures and in the epithelial component of 35/57 PTs (61.4%). No correlation was observed between *MED12* mutation status and a particular MED12 pattern in the stromal component of PTs. (Table 4). In the epithelial component, ER nuclear expression was present in 42/57 PTs (73.7%) and PR nuclear expression was present in 55/57 PTs (96.5%). No ER and PR nuclear expression was identified in the stromal component. No correlation was observed between *MED12* mutation status and a particular ER and PR pattern in the epithelial component of PTs. Our immunohistochemistry results also failed to demonstrate any significant correlation between *MED12* mutation status and Ki67 index (Table 4).

**Table 4:** Associations between *MED12* mutation status and pathological features of 57 PTs

	Total population (%)	*MED12* wild type PTs (%)	*MED12* mutated PTs (%)	*p*-value^a^
***Total***	57 (100)	32 (56.1)	25 (43.9)	
***ER epithelial expression***				
Negative (0–1)	15 (26.3)	11 (73.3)	4 (26.7)	0.12 (NS)
Positive (> 2)	42 (73.7)	21 (50.0)	21 (50.0)	
***PR epithelial expression***				
Negative (0–1)	2 (3.5)	2 (100)	0 (0)	0.58 (NS)
Positive (> 2)	55 (96.5)	30 (54.5)	25 (45.5)	
***MED12 stromal expression***				
Negative (0–1–2)	11 (19.3)	6 (54.5)	5 (45.5)	0.83 (NS)
Positive (> 3)	46 (80.7)	26 (56.5)	20 (43.5)	
***Ki67 index***				
Negative (0–19%)	39 (68.4)	21 (53.8)	18 (46.2)	0.61 (NS)
Positive (> 19%)	18 (31.6)	11 (61.1)	7 (38.9)	
***Transgelin stromal expression***				
Negative (0–1)	41 (71, 9)	27 (84.3)	14 (56)	0.018
Positive (> 2)	16 (28)	5 (15.6)	11 (44)	

a Chi-square test.

NS: not significant.

In order to obtain further insight into *MED12* mutations in PTs, qRT-PCR was used to evaluate mRNA expression of a large number of selected genes from the 37 *MED12*-WT and 30 *MED12*-mutated PTs. The expression levels of 44 genes involved in various cellular and molecular phenomena associated with tumorigenesis, as well as genes involved in various steps of the *MED12* signaling pathway in human embryogenesis and other types of *MED12*-associated cancers reported in the literature were assessed [19, 20, 21, 22]. These genes encode proteins involved in cell cycle regulation (*n* = 6), apoptosis (*n* = 1), and EMT (*n* = 4), growth factor receptors (*n* = 3) and nuclear receptors (*n* = 3), genes involved in *TGFB* (*n* = 4), *WNT* (*n* = 8), Hedgehog (*n* = 3) and *REa* (*n* = 7), signaling pathways, and stem cell markers (*n* = 5).

**Figure 3:**
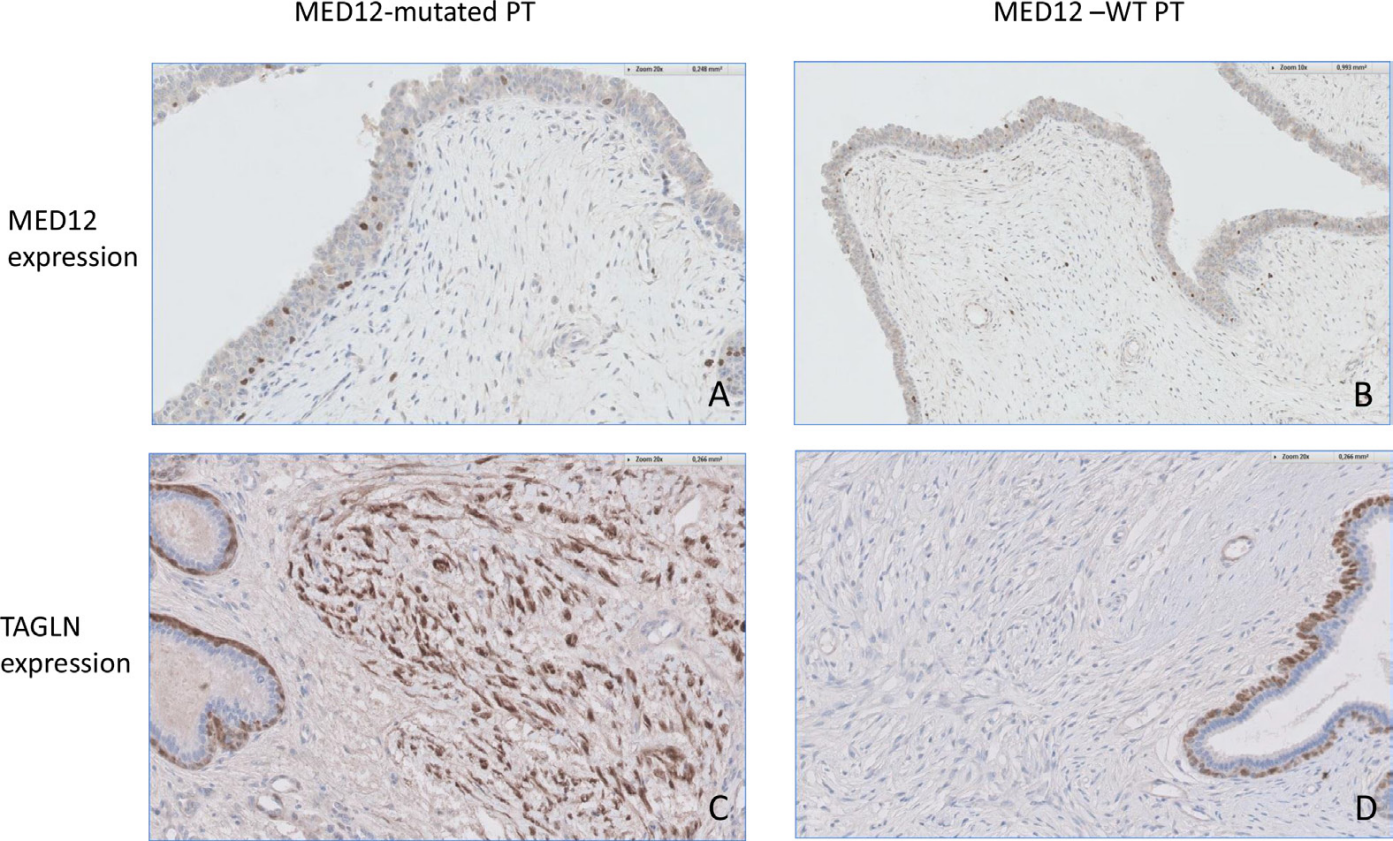
(**A**) Focal MED12 nuclear expression in the stromal component of a *MED12*-mutated PT. × 20 objective. (**B**) Focal *MED12* nuclear expression in the stromal component of a *MED12*-WT PT. × 20 objective. (**C**) High TAGLN expression in the stromal component of a *MED12*-mutated PT. × 20 objective. Note the TAGLN expression by myoepithelial cells. (**D**) Low TAGLN expression in the stromal component of a *MED12*-WT PT. ×20 objective. Note the TAGLN expression by myoepithelial cells.

The genes significantly associated with *MED12* mutations were mainly involved in the *WNT* (*PAX3, WNT3A, AXIN2*), *TGFB* (*TAGLN, TGFBR2, CTGF)* and *THRA (RXRA, THRA,)* signaling pathways (Supplementary Table S2). Expressions of genes involved in other signaling pathways (particularly the *REa* signaling pathway) were either not correlated or only weakly correlated (a weak tendancy is found for *ESR1, FOXA1, CA12*) with *MED12* mutation status. TAGLN expression was evaluated by immunohistochemistry in this series of 57 PTs with known *MED12* status (Figure 3), which confirmed the statistically significant association between high TAGLN protein expression and *MED12*-mutated tumors compared to *MED12*-WT tumors (*p* = 0.018) (Table 4).

## DISCUSSION

This study confirmed the high prevalence of *MED12* somatic mutations in 49.4% (41/83) of PTs and 70% (7/10) of FAs. The mutation frequency in FAs and PTs was similar to that recently reported: *MED12* mutations in 67% to 70% of PTs [5, 6, 11] and 59% to 85% of FAs [6, 10, 11] and. The low level of *MED12* mutations in fibromatosis indicates that this gene is not a major mutational event in this tumor type.

A high prevalence of *MED12* mutations was identified in 14/24 (58.3%) benign PTs and 19/30 (63.3%) borderline PTs. In contrast, *MED12* exons 1 and 2 mutations were significantly less frequent in malignant PTs (8/29 – 27.6%) (*p* = 0.0036), which is consistent with two recent studies describing *MED12* somatic mutations in 88%, 78% and 8% of benign, borderline and malignant PTs, respectively, in a series of 57 PTs [10] and in 80% of benign and borderline PTs and 40% of malignant PTs in a series of 15 PTs [5]. In agreement with these two reports, the mutations identified in the present study were mostly substitutions (51.2%) and insertion-deletion (48.8%) mutations. All exons 1 and 2 mutations identified in our series have been previously reported.

We investigated temporal heterogeneity in this series of PTs. Overall, in 70% (7/10) of cases, the *MED12* mutation status (mutation or WT) remained the same in recurrent lesions. Three (50%) of the 6 *MED12* mutation-positive primary tumors harbored different mutations in the recurrent lesions, highlighting the temporal heterogeneity of these tumors. This change in mutation status could be explained by the formation of a new clonal proliferation or a clone that became predominant over the others. This temporal heterogeneity also raises the possibility that these tumors are more likely to be new primary lesions rather than recurrences, as it would be unlikely for a new *MED12* mutation to emerge in a tumor that already harbors a *MED12* mutation. Temporal heterogeneity of PTs should be taken into account in treatment options and recurrent tumors should be screened for new mutations. In contrast, PTs did not display any spatial heterogeneity.

A possible correlation between *MED12* exon 2 mutation status and outcome was investigated in this series of 83 PTs. The presence of a *MED12* mutation was not associated with patient outcome in the overall population (*p* = 0.51) or in the subpopulation of 30 borderline PTs (*p* = 0.74, data not shown).

Among borderline and malignant tumors, the genomic profiles of the 26 *MED12*-mutated PTs and the 27 *MED12*-WT tumors have the same median number of chromosomal number alterations CNAs = 3) (Kruskal Wallis's H test, *p* = 0.36). The eight most frequent recurrent imbalances encountered in our series of 53 PTs were not significantly associated with *MED12* mutation status, even within each group of borderline and malignant PTs (data not shown).

No correlation was observed between *MED12* mutations and MED12 expression in the stromal component of PTs, ER/PR expression in the epithelial component of PTs and Ki67 index as a marker of cell proliferation.

Our results showed a significant relationship between *MED12* and eight genes: genes of *WNT* (*PAX3 WNT3A, AXIN2*), *TGFB (TAGLN, TGFBR2*, CTGF) and *THRA (RXRA ,THRA)* signaling pathways. These results confirm that *MED12* has a role to be recruited to β-catenin- responsive promoters in a β-catenin-dependent manner to activate transcription in response to Wnt signaling, via PAX3, WNT3a and AXIN2 [20, 21].

These results also confirm the interaction between *MED12* and *TGFbR2* [22]. *MED12* is active in the cytoplasm, where it negatively regulates *TGFbR2* via a physical interaction. *MED12* suppression therefore results in activation of TGF-bR signaling, which is both necessary and sufficient for drug resistance [22, 23]. Further investigations are necessary to study the intriguing interaction between *MED12* signaling pathways and *THRA* and *RXRA* nuclear receptors [24]. It is noteworthy that *RARA*, recently identified as being frequently mutated in PTs [6], was not differentially expressed between *MED12*-mutated tumors and *MED12*-WT tumors in our series.

In contrast with Tan [6] and Lim [10], who found that genes upregulated in *MED12*-mutant FAs were associated with ER+ breast cancers, estrogen stimulus in ER+ breast cancer cells and activated estrogen signaling, we did not find any link between *MED12* and *ESR1* signaling pathways.

In conclusion, the present study confirms that *MED12* exon 1 and 2 mutations are frequent oncogenic events in breast PTs. *MED12* mutation is the leading recurrent oncogenic mechanism demonstrated in both benign and malignant breast PTs, but is less frequent in malignant PTs, suggesting that genetic or epigenetic alterations other than *MED12* may play a role in tumor aggressiveness and progression. The high frequency and similar patterns of *MED12* mutations in FAs and various grades of PTs imply that *MED12* mutation is a common and early pathological event in breast fibroepithelial tumors. Our study shows that PTs present temporal heterogeneity of *MED12* mutation status (with consequences for potential targeted cancer therapies), that MED12 nuclear expression in the epithelial component of phyllodes tumors is associated with *MED12*-mutated tumors, and that, among all genomic alterations described in PTs, no alteration is significantly associated with *MED12*-WT tumors

We have also demonstrated a correlation between *MED12* and *TGFB, THRA* and *WNT* signaling pathways, and the absence of correlation with ER*a* pathways. Further studies must be conducted to characterize the biological roles of *MED12* oncoprotein and assess the possibility of therapeutic inhibition of *MED12*-mediated oncogenic consequences.

## MATERIALS AND METHODS

### Case selection

One hundred and eighteen breast tumors from 104 female patients were analyzed: 97 PTs (83 primary tumors, 13 recurrences and 1 metastasis), 10 FAs and 11 cases of fibromatosis extracted from our institution's archives. The study was approved by the Institut Curie Institutional Review Board. All cases were centrally reviewed by two pathologists (ML and PT). PTs were classified into three categories, benign, borderline, and malignant according to the 2012 WHO classification (1): Grading was based on semiquantitative evaluation of the following criteria in the stromal component: stromal atypias, mitotic count per 10 high-power fields, overgrowth, cellularity, and appearance of tumor margins (well-defined or invasive). In addition, the mitotic count cut-off was less than 5 per 10 high-power fields for benign, 5–9 for borderline, 10 or more for malignant PTs. Twenty-four of the 83 primary phyllodes tumors were classified as benign, 30 were classified as borderline and 29 were classified as malignant. The age of the patients ranged from 10 to 74 years (mean age: 43.8 years, median age: 47.0 years). Tumor size ranged from 12 to 220 mm (mean: 60.1 mm and median: 50.0 mm). Follow-up was available for all tumors with a median follow-up of 38.4 months. Clinicopathological data (age, tumor size, histological grade, follow-up, second events such as recurrences or metastasis) were collected (Table 1).

### DNA extraction from fresh frozen and formalin-fixed and paraffin-embedded tissue

We used the Qiagen DNeasy Tissue kit and protocols for fresh frozen (*n* = 66) and formalin-fixed and paraffin-embedded tissues (*n* = 42). Tumor areas were microdissected to enrich for neoplastic cells (> 80%). For formalin-fixed and paraffin-embedded tissue, 6 × 6-*μ*m-thick tissue sections were collected in 1.5 mL Eppendorf tubes. Paraffin was dissolved in xylene and removed. Tissue samples were then lysed under denaturing conditions with proteinase K digestion at 56 ± 1°C for 1 h, followed by incubation at 90°C to reverse formalin cross-linking. DNA was purified by column purification with a filter membrane and stored at −20°C until use.

### PCR amplification and Sanger sequencing

For PCR amplification, 20 ng of genomic DNA was used. Primers used were: 5′- TCGGGATCTTGAGCTACGAACA-3′ (forward) and 5′-AGCCGTCAGTGCCTCCTCCTA-3′ (reverse) for amplification of *MED12* exon 1 (160 pb PCR product); and: 5′- AAAAAACAACTAAACGCCGCTT-3′ (forward) and 5′-TGT CCC TAT AAG TCT TCC CAA CC-3′ (reverse) for amplification of *MED12* exon 2 (196 pb PCR product). The reactions were then run on 3500xL and 3130 Genetic Analyzers (Applied Biosystems, Foster City, CA, USA). Mutations/variations were analyzed by Sequencing analysis software.

### Determination of copy number alterations

For 53 PTs [borderline (*n* = 30) and malignant (*n* = 23)], 700 ng of tumor DNA were analyzed with Human CNV370 BeadChip (Illumina Inc., San Diego, CA), containing 370,000 SNP markers, with a mean spacing of 7.9 kb, as previously described [25]. Hybridizations were performed by IntegraGen (Evry, France), according to instructions provided by Illumina. Fluorescence signals were imported into BeadStudio software (Illumina) and normalized.

Gains, amplifications, and losses were defined using the Illumina SNP/CNV analysis pipeline software developed in INSERM U900 at Institut Curie. VAMP software was used to visualize and analyze copy number alterations. To determine the overall profiles of defined sets of tumors for each probe, the fraction of tumors with gains and losses over the dataset was computed and displayed in the FrAGL (Frequency of Amplicons, Gains and Losses) view. Further descriptions can be found at: http://bioinfo-out.curie.fr/vamp/doc/userManual.

### RNA extraction

Total RNA was extracted from frozen breast tumor samples (*n* = 67) using the acid-phenol guanidium method. The quantity of RNA was assessed by using an ND-1000 NanoDrop Spectrophotometer with its corresponding software (Thermo Fisher Scientific Inc., Wilmington, DE). RNA quality was determined by electrophoresis through agarose gel and ethidium bromide staining. The 18S and 28S RNA bands were visualized under ultraviolet light.

### RT-PCR

Quantitative values were obtained from the cycle number (Ct value) at which the increase in the fluorescence signal associated with exponential growth of PCR products started to be detected by the laser detector of the ABI Prism 7900 sequence detection system (Perkin–Elmer Applied Biosystems, Foster City, CA, USA), using the PE Biosystems analysis software (Perkin Elmer Applied Biosystems) according to the manufacturer's manuals. The precise amount of total mRNA added to each reaction mix (based on optical density) and its quality (i.e., lack of extensive degradation) are both difficult to assess. We therefore also quantified *TBP* gene transcripts (Genbank accession NM_003194) encoding the TATA box-binding protein (a component of the DNA-binding protein complex TFIID) as an endogenous RNA control and normalized each sample on the basis of its *TBP* content. We selected *TBP* as endogenous control, because of the moderate prevalence of its transcripts and the absence of any known *TBP* retropseudogenes (retropseudogenes lead to coamplification of contaminating genomic DNA and consequently interfere with RT–PCR, despite the use of primers in separate exons) [26]. Results are expressed as N-fold differences in target gene expression relative to the *TBP* gene and termed ’N_target_=2^DCT^’, where the ΔCt value of the sample was determined by subtracting the Ct value of the target gene from the Ct value of the *TBP* gene.

Primers for *TBP* and target genes were chosen by using Oligo 6.0 software (National Biosciences, Plymouth, MN, USA). We scanned the dbEST and nr databases to confirm the total gene specificity of the nucleotide sequences chosen for the primers and the absence of single-nucleotide polymorphisms. To avoid amplification of contaminating genomic DNA, one of the two primers was placed at the junction between two exons. Agarose gel electrophoresis was used to verify the specificity of PCR amplicons. The conditions of cDNA synthesis and PCR were as previously described [27].

### Immunohistochemical analysis

Immunohistochemistry was performed on representative 3-μm-thick whole tissue sections from 57 tumors of our series of 87 PTs, mounted on SUPERFROST slides (Thermo Scientific, Braunschweig, Germany). Tissue sections were obtained from formalin-fixed paraffin-embedded (FFPE) blocks. After rehydration and antigen retrieval in citrate buffer (10 mM, pH 6.1), tissue sections were stained for estrogen receptor (ER, clone 6F11, Novocastra at 1/50 dilution), progesterone receptor (PR, clone 16, Novocastra, 1/600), Ki67 (Clone MIB1, DAKO, Glostrup, Denmar 1/200), transgelin -TAGLN (rabbit polyclonal, GeneTex, 1/200) and MED12 (rabbit polyclonal, Origene, 1/200). Revelation of staining was performed using the BOND Polymer Refine Detection Kit (Leica Biosystems Newcastle Ltd, United Kingdom) with DAB as chromogen. Positive and negative controls were included in each slide run.

Immunostains were analyzed using the Allred scoring system that combines the staining intensity and the percentage of stained cells (intensity score 0–3 + % score 0–5) [27]. For each case, the score was assessed separately for membranous, cytoplasmic and nuclear reactivity. Epithelial and spindle cell components were evaluated separately. For MED12, an Allred score of > 3 was considered to be positive. For ER and PR, an Allred score of > 1 was considered to be positive. The Ki67 index was assessed semiquantitatively by estimating, at × 40 magnification, the percentage of positive neoplastic nuclei within the area of highest positivity chosen after scanning the entire tumor surface at low power (× 10 objective). All nuclei with homogeneous staining, even with only weak staining or nucleolar staining, were interpreted as positive. A cut-off of 20% was used to define tumors with a high Ki67 score.

### Statistical analysis

The distributions of MED12 RNA and protein levels, and other target RNA and protein levels were characterized by their median values and ranges. Relationships between tumor changes (expressed as mutational or expression status) and clinical, pathological and laboratory parameters were estimated with the Chi^2^ and Kruskal-Wallis tests. Disease-free survival (DFS) was determined as the interval between diagnosis and detection of the first second event (recurrence or metastasis). Survival distributions were estimated by the Kaplan-Meier method and the significance of differences between survival rates was determined with the log-rank test [29]. *P* values < 0.05 were considered significant.

## SUPPLEMENTARY MATERIALS FIGURES AND TABLES





## Abbreviations

PTs: phyllodes tumors
ER: oestrogen receptor
PR: progesterone receptor.
