# Supplementary material for: MED12 mutations in breast phyllodes tumors: evidence of temporal tumoral heterogeneity and identification of associated critical signaling pathways
Source: Oncotarget. 2016 Oct 31;7(51):84428–38. doi: 10.18632/oncotarget.12991 (PMC5356671; doi:10.18632/oncotarget.12991)
Supplement: Supplementary file 1 [file oncotarget-07-84428-s001.pdf]

MED12 mutations in breast phyllodes tumors: evidence of temporal tumoral heterogeneity and identification of associated critical signaling pathways

Supplementary Materials

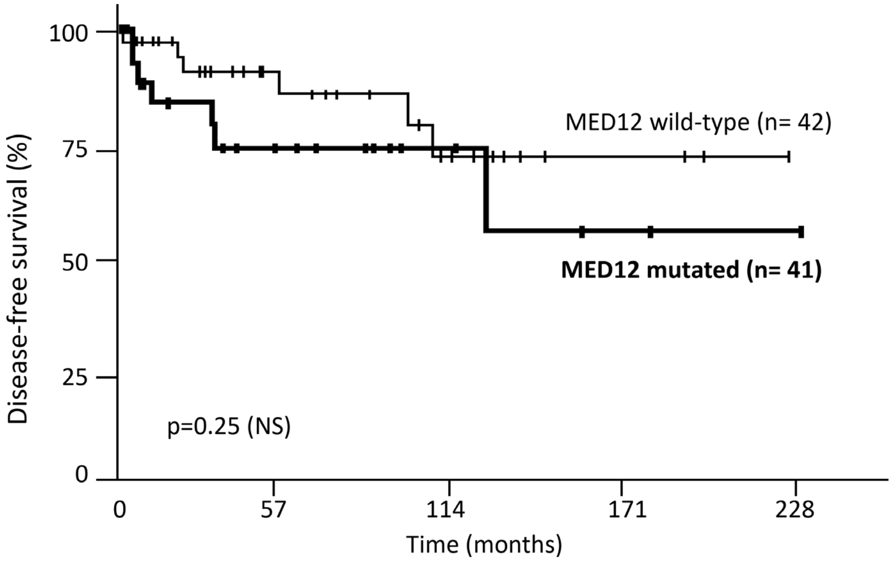

Supplementary Figure S1: Disease-free survival curves for the overall PT population. Disease-free survival curves of patients with *MED12*-WT and *MED12* -mutated tumors.

Supplementary Table S1: Tabular summary of *MED12* exon 1 and 2 mutations in PTs, including their observed frequencies, in the series of 83 PTs

Mutation Type	CDS mutation	AA mutation	Mutation location	MED12 mutated tumors	
				Number	%
Substitution	c.131G > A	p.Gly44Asp	exon 2	8	9,6%
	c.131G > T	p.Gly44Val	exon 2	5	6,0%
	c.130G > A	p.Gly44Ser	exon 2	4	4,8%
	c.130G > T	p.Gly44Cys	exon 2	1	1,2%
	c.131G > C	p.Gly44Ala	exon 2	1	1,2%
	c.130G > C	p.Gly44Arg	exon 2	1	1,2%
	c.107T > G	p.Leu36Arg	exon 2	1	1,2%
Total substitutions				***21***	***25,3%***
Deletion	c.94_96delAAG	p.Lys32del	exon 1	1	1,2%
	c.82_99del18 + c.80A > C	p.Asp28_ Glu33del + p.27Gln > Pro	exon 1	1	1,2%
	c.100-5_129del	p.?	exon 2	1	1,2%
	c.117_149del	p.Leu39_ Ala50delinsPhe	exon 2	1	1,2%
	c.100-5_149del	p.?	exon 2	1	1,2%
	c.122_148del	p.Val41_Pro49del	exon 2	2	2,4%
	c.121_153del	p.Val41_Val51del	exon 2	1	1,2%
	c.120_147delinsA	p.Asn40_ Pro49delinsLys	exon 2	1	1,2%
	c.100_144del	p.Asp34_ Gln48del	exon 2	1	1,2%
	c.120_142del	p.Asn40Lysfs*8	exon 2	1	1,2%
	c.119_149delinsT	p.Asn40_ Ala50delinsIle	exon 2	1	1,2%
	c.132_158del	p.Phe45_Gly53del	exon 2	1	1,2%
	c.122_150delinsCTA	p.Val41Alafs*6	exon 2	1	1,2%
	c.118_153del	p.Asn40_Val51del	exon 2	1	1,2%
	c.123_152del	p.Lys42_Val51del	exon 2	1	1,2%
	c.100-3_138del	p.?	exon 2	1	1,2%
	c.107_122del	p.Leu36Glnfs*56	exon 2	1	1,2%
	c.120_128del	p.Asn40_ Gln43delinsLys	exon 2	1	1,2%
	c.125_145del	p.Lys42_ Pro49delinsThr	exon 2	1	1,2%
Total deletions				***20***	***24,1%***
**Total Mutations**				41	49,4%

Abbreviations: CDS: coding sequence; AA: amino acid.

Supplementary Table S2: Links between target gene mRNA expressions and *MED12* mutation status in a series of 67 PTs. See Supplementary_Table_S2.
