# Supplementary material for: MED12 mutations in breast phyllodes tumors: evidence of temporal tumoral heterogeneity and identification of associated critical signaling pathways
Source: Oncotarget. 2016 Oct 31;7(51):84428–38. doi: 10.18632/oncotarget.12991 (PMC5356671; doi:10.18632/oncotarget.12991)
Supplement: Supplementary file 2 [file oncotarget-07-84428-s002.doc]

| Supplemental table 2: Links between target gene mRNA expressions and *MED12* mutation status in a series of 67 PTs. | | | | | |  |
| --- | --- | --- | --- | --- | --- | --- |
|  | | | | | |  |
| **GENES** | **Normal breast tissues (n=10)** | ***MED12* wild-type PTs (n=37)** | ***MED12* mutated PTs (n=30)** | ***p*-value** ^a^ | **ROC-AUC** ^b^ |  |
|  |  |  |  |  |  |  |
|  |  |  |  |  |  |  |
| ***Cell cycle regulation (n=6)*** |  |  |  |  |  |  |
| **MKI67** | 1.00 (0.45-3.45) ^c^ | 12.0 (0.23-39.9) ^c^ | 10.8 (1.73-32.6) ^c^ | 0.97 (NS) | 0.498 |  |
| **CCND1** | 1.00 (0.58-1.33) | 1.26 (0.19-2.90) | 1.37 (0.04-2.91) | 0.99 (NS) | 0.500 |  |
| **RB1** | 1.00 (0.54-1.12) | 1.10 (0.07-1.74) | 1.09 (0.10-2.80) | 0.47 (NS) | 0.449 |  |
| **MDM2** | 1.00 (0.8-1.17) | 1.05 (0.33-2.31) | 1.08 (0.59-1.74) | 0.99 (NS) | 0.500 |  |
| **CDKN2A/P16** ^d^ | 0.00 (0.00-6.48) | 3.93 (0.00-57.2) | 2.78 (0.00-21.2) | 0.33 (NS) | 0.430 |  |
| **CDKN2A/ARF** | 1.00 (0.00-2.53) | 2.23 (0.13-41.2) | 1.47 (0.18-9.09) | 0.075 (NS) | 0.373 |  |
| ***Apoptosis (n=1)*** |  |  |  |  |  |  |
| **BBC3** | 1.00 (0.56-1.43) | 1.44 (0.30-3.09) | 1.40 (0.18-2.84) | 0.97 (NS) | 0.503 |  |
| ***EMT (n=4)*** |  |  |  |  |  |  |
| **ZO-1** | 1.00 (0.44-2.62) | 8.38 (0.09-145) | 5.64 (1.63-73.0) | 0.69 (NS) | 0.471 |  |
| **CDH1** | 1.00 (0.58-2.18) | 0.90 (0.00-4.37) | 1.25 (0.00-2.40) | 0.22 (NS) | 0.587 |  |
| **CDH2** | 1.00 (0.33-1.32) | 1.18 (0.36-3.01) | 1.07 (0.33-2.23) | 0.14 (NS) | 0.393 |  |
| **VIM** | 1.00 (0.41-1.80) | 0.41 (0.14-1.24) | 0.47 (0.25-1.37) | 0.099 (NS) | 0.618 |  |
| ***Growth factor receptors (n=3)*** |  |  |  |  |  |  |
| **MET** | 1.00 (0.41-1.58) | 0.31 (0.03-41.1) | 0.43 (0.01-2.09) | **0.022** | 0.664 |  |
| **EGFR** | 1.00 (0.23-1.43) | 1.27 (0.22-21.3) | 0.97 (0.17-7.89) | 0.10 (NS) | 0.383 |  |
| **IGF1R** | 1.00 (0.11-1.65) | 1.65 (0.05-4.46) | 1.32 (0.07-3.75) | 0.76 (NS) | 0.478 |  |
| ***Nuclear receptors (n=3)*** |  |  |  |  |  |  |
| **RXRA** | 1.00 (0.37-2.39) | 1.38 (0.70-2.57) | 1.02 (0.80-3.83) | **0.00035** | 0.245 |  |
| **THRA** | 1.00 (0.66-1.35) | 0.62 (0.20-1.67) | 0.44 (0.12-1.29) | **0.0094** | 0.314 |  |
| **RARA** | 1.00 (0.38-1.26) | 2.04 (0.62-3.32) | 1.99 (0.46-11.56) | 0.95 (NS) | 0.495 |  |
| ***TGFB pathway (n=4)*** |  |  |  |  |  |  |
| **TAGLN** | 1.00 (0.69-1.47) | 0.46 (0.01-1.31) | 0.81 (0.05-1.78) | **0.00049** | 0.749 |  |
| **TGFBR2** | 1.00 (0.76-1.66) | 0.49 (0.20-1.03) | 0.39 (0.11-0.72) | **0.0083** | 0.311 |  |
| **CTGF** | 1.00 (0.51-2.68) | 1.13 (0.15-8.50) | 1.97 (0.24-10.1) | **0.023** | 0.662 |  |
| **CYR61** | 1.00 (0.26-1.91) | 0.36 (0.14-4.38) | 0.65 (0.08-2.21) | 0.50 (NS) | 0.549 |  |
| ***WNT pathway (n=8)*** |  |  |  |  |  |  |
| **PAX3** | 1.00 (0.00-7.52) | 15.3 (1.93-117) | 3.84 (0.05-125) | **0.000088** | 0.220 |  |
| **WNT3A** ^d^ | 0.00 (0.00-2.33) | 0.35 (0.00-4.22) | 0.00 (0.00-2.69) | **0.00074** | 0.259 |  |
| **AXIN2** | 1.00 (0.63-1.52) | 1.23 (0.14-2.48) | 0.94 (0.15-2.29) | **0.018** | 0.331 |  |
| **MMP7** | 1.00 (0.25-2.25) | 0.25 (0.00-7.76) | 0.92 (0.00-3.07) | 0.11 (NS) | 0.615 |  |
| **LEF1** | 1.00 (0.70-1.70) | 3.56 (0.41-11.3) | 3.03 (1.08-13.5) | 0.43 (NS) | 0.443 |  |
| **WNT5A** | 1.00 (0.42-1.82) | 2.52 (0.58-5.67) | 2.56 (1.04-13.7) | 0.31 (NS) | 0.572 |  |
| **DKK1** | 1.00 (0.00-3.18) | 2.08 (0.34-63.0) | 2.11 (0.57-34.0) | 0.73 (NS) | 0.476 |  |
| **DKK3** | 1.00 (0.58-1.51) | 1.01 (0.10-7.73) | 1.06 (0.21-3.56) | 0.91 (NS) | 0.508 |  |
| ***Hedgehog pathway (n=3)*** |  |  |  |  |  |  |
| **CREBBP** | 1.00 (0.60-1.29) | 1.17 (0.47-2.24) | 0.95 (0.57-2.75) | **0.0077** | 0.309 |  |
| **GLI2** | 1.00 (0.48-1.59) | 4.79 (0.44-9.78) | 3.74 (0.92-24.9) | 0.14 (NS) | 0.395 |  |
| **GLI3** | 1.00 (0.34-1.36) | 1.06 (0.30-1.80) | 0.96 (0.18-1.60) | 0.42 (NS) | 0.443 |  |
| ***Stem cell (n=5)*** |  |  |  |  |  |  |
| **NANOG** ^d^ | 0.00 (0.00-2.82) | 5.61 (0.00-259) | 8.14 (0.00-653) | 0.81 (NS) | 0.483 |  |
| **SOX9** | 1.00 (0.44-1.96) | 0.53 (0.04-1.77) | 0.51 (0.00-1.26) | 0.93 (NS) | 0.506 |  |
| **SOX2** | 1.00 (0.00-2.96) | 0.51 (0.10-16.8) | 0.47 (0.13-147) | 0.65 (NS) | 0.468 |  |
| **NEFL** | 1.00 (0.46-2.67) | 1.12 (0.24-12.1) | 1.24 (0.68-49.7) | 0.16 (NS) | 0.601 |  |
| **BMP4** | 1.00 (0.23-2.42) | 0.92 (0.19-3.01) | 0.68 (0.21-1.48) | 0.051 (NS) | 0.360 |  |
| ***Era-inducible genes (n=7)*** |  |  |  |  |  |  |
| **ESR1** | 1.00 (0.36-2.62) | 0.87 (0.00-7.80) | 1.89 (0.00-13.9) | **0.030** | 0.655 |  |
| **PGR** | 1.00 (0.28-2.16) | 1.04 (0.00-5.55) | 1.05 (0.00-5.14) | 0.96 (NS) | 0.504 |  |
| **FOXA1** | 1.00 (0.27-1.49) | 0.78 (0.00-2.72) | 1.12 (0.00-2.77) | **0.037** | 0.649 |  |
| **CYP2B6** | 1.00 (0.31-3.48) | 1.95 (0.05-24.3) | 2.50 (0.04-49.8) | 0.57 (NS) | 0.541 |  |
| **CA12** | 1.00 (0.75-2.09) | 0.89 (0.00-4.0) | 1.44 (0.06-3.04) | **0.040** | 0.647 |  |
| **IL6ST** | 1.00 (0.00-10.11) | 4.44 (0.23-41.0) | 3.38 (0.22-25.6) | 0.48 (NS) | 0.450 |  |
| **STC2** | 1.00 (0.58-2.32) | 0.90 (0.01-3.73) | 1.18 (0.05-3.34) | 0.099 (NS) | 0.618 |  |
|  |  |  |  |  |  |  |
|  |  |  |  |  |  |  |
| ^a^ Kruskal-Wallis H Test. |  |  |  |  |  |  |
| ^b^ ROC (Receiver Operating Characteristics) - AUC (Area Under Curve) analysis. | | | |  |  |  |
| ^c^ Median (range) of gene mRNA levels; mRNA values of the samples were normalized so that the median of the 10 normal breast tissue mRNA values was equal to 1. | | | | | | |
| ^d^ mRNA values of the samples were normalized so that a Ct value of 35 was equal to 1. | | | |  |  |  |
